# Supplementary material for: High-Content C. elegans Screen Identifies Natural Compounds Impacting Mitochondria-Lipid Homeostasis and Promoting Healthspan
Source: Cells. 2021 Dec 29;11(1):100. doi: 10.3390/cells11010100 (PMC8750055; doi:10.3390/cells11010100)
Supplement: Supplementary file 1 [file cells-11-00100-s001.zip › cells-1501642-supplementary/cells-1501642-SM PROOF DONE .pdf]

Supplementary Information

Supplementary Tables

Table S1: List of natural compounds tested in the screening

Name	Chemical nature	Natural Source(s)	Purity	Biological activity	Effect on gfp/size	
**Helenalin**	Sesquiterpene lactone	Plant, Arnica montana	>95%	Antiinflammatory [61, 62]		
**Hexaprenylhydrochinon**	Methylated derivative	Red Sea sponge, Sarcotragus muscarum	>95%	inhibitor of retroviral reverse transcriptases, & cellular DNA polymerases [63]	-	
**Homosekikaic acid**	Natural acid	Lichen, Ramalina farinacea	>95%	Antimicrobial and antioxidant activity [64]	-	-
**2-Hydroxy-4-methoxyphenylacetonitrile**	---------- information not available----------				-	
**Hydroxysydonic acid**	Phenolic compund	Fungus, Aspergillus sydowii	>95%	Antibacterial [65]		
**Hyperoside**	Phenolic flavonoid	Flower, Geranium molle	>95%	Antibacterial [66, 67]		
**Indol-3-carboxylic acid**	Flavon glycoside	Callyspongia Biru	>95%	Antifungal [68, 69]		
**Isobavachalcone**	Flavonoids, Phytochemical	Chinese herb medicine Psoralea corylifolia	>95%	Immunoregulation, antioxidation [70, 71]	+	-
**Isovitexin**	Apigenin-6-C-glucoside	Vica Faba Vigna Radiata	>95%	Antioxidant activity, antiinflammatory and anticancer [72-74]	-	
**Kaempferol-3-rutinoside**	Flavonol glycoside	Plant, Scorzonera Radiata	>95%	inhibitor of α-glucosidase [77]	-	
**Kaempferitrin**	Flavonoid glycoside	Tagetes erecta L. reinforces and Bauhinia forficata leaves	>95%	Anxiolytic and sedative- effect [78, 79]		
**Kaempferol 3-O-β-D-glucopyranosyl (1-4) α-L-rhamnopyranosyl-7-O-α-L-rhamnopyranoside**	Flavonol-glycosides derivate	Shrub, Olax manni	>95%	Antiinflamatory [80-82]	-	-
**Kahalalide F**	Cyclic-peptide	Mollusks, eg. Elysia rufescens and Elysia grandifolia	>95%	Anticancer, antibacterial, antifungal [52-55]	-	-
**Kojic acid**	Chelating agent	Lecytophora sp. Alstonia scholaris	>95%	Antifungal [83]	-	
**Kuanoniamine D**	Pyridoacridine alkaloid	Micronesian sponge Oceanapia	>95%	Anticancer [84-88]	-	-
**Longamide B**	Alkaloid	marine sponge Acanthostylotella spp.	>95%	Anticancer [89]		
**Luffariellolid**	Sesterterpene	marine sponge, Acanthodendrilla sp.	>95%	Antiinflammatory, anticancer [90, 91]	-	-

**Lupeol**	Pentacyclic lupane-type triterpene	Plant, Dianthus versicolor	>95%	Anticancer [92, 93]	+	-
**Lutein**	Carotenoid	Aglaia oligophylla Microalgae, Leaves of Ilex guayusa, Flowers	>95%	Antioxidant [94-97]		-
**Macrosporin**	Mycotoxin	Pleospora sp.	>95%	Weak antibacterial [98-101]	+/-	-
**Manzamine A**	Alkaloid	marine Sponge, Achantostrongylophora ingens	>95%	Anticancer, antibacterial, antiviral [102-105]	-	-
**Manzamine F**	Ketone	marine sponge, Achantostrongylophora ingens	>95%	Anticancer [106]	-	-
**8-OH-manzamine A**	Alkaloid	marine sponge, Achantostrongylophora ingens	>95%	Antimalarial [106]	-	-
**(-)-Matairesinol**	Polyphenolic lignans	Plant, Dianthus versicolor	>95%	Anti-estrogen receptor activity [107, 108]	-	
**Mauritamide B**	Alkaloid	Marine sponge, Agelas nsp.	>95%	Antimicrobial [109]		

Compounds of marine origin are highlighted by rows with gray-background. Purity was assessed by HPLC. (-) Indicates a decrease in GFP intensity or in body size while (+) indicates an increase in GFP intensity or in body size compared to control animals.

Table S2: Lifespan's statistics

C elegans strain	Treatment	Lifespan change (%)[a]	P-value vs control[b]	Sample size/ n trials (censored)
**N2 (wild-type)**	control (DMSO)			240/4 (72)
**N2 (wild-type)**	lutein 100 µM	+15.6	0.0001	240/4 (62)
**N2 (wild-type)**	kahalalide 0.5 µM	+10.1	0.0155	120/2 (29)
**VC228 *nlg-1*(ok259)**	control (DMSO)		0.003	120/2 (13)
**VC228 *nlg-1*(ok259)**	lutein 100 µM	-4	0.52	120/2 (19)

Table Legend

[a] % increase normalized mean lifespan compared to its control; [b] Kaplan-Meier survival analysis, Log-rank test against control.

Supplementary Figures

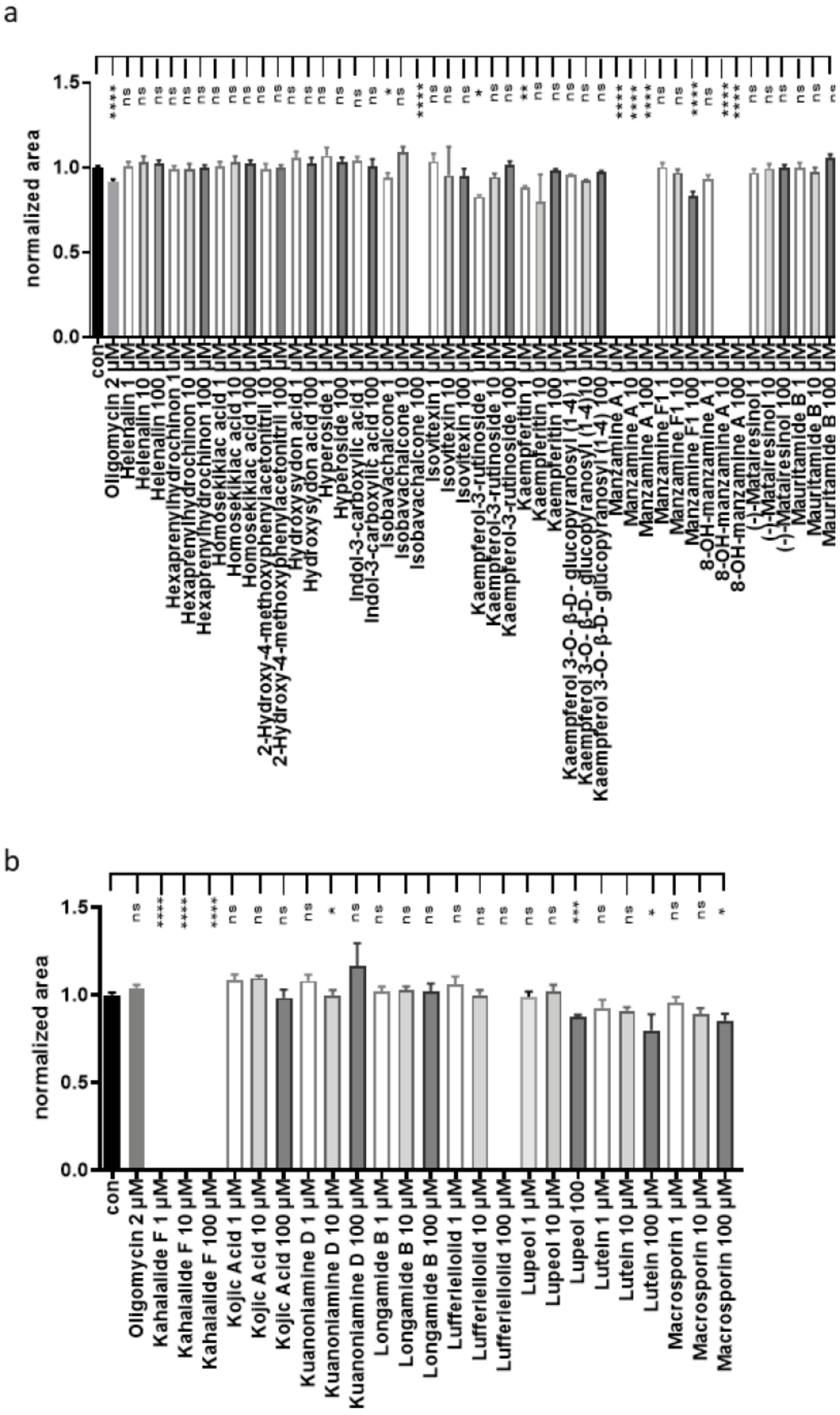

Figure S1. Animals' size quantified with the Cellomic ArrayScan reader in liquid culture. In **a)** the *C. elegans* strain used for the screening was a transcriptional reporter for *gst-4* and in **b)** a transcriptional reporter for *hsp-6*. The results highlight few compounds with the ability to significantly alter the size of the nematodes. The area was normalized to 1 for control worms, treated with 1% DMSO. Bar graphs represent means ± SEM. Asterisks (*) denote significant differences vs control, unpaired t-test. *p-value <0.05 ** p value < 0.01, ***p-value < 0.001, ****p-value < 0.0001; ns stands for not significant.

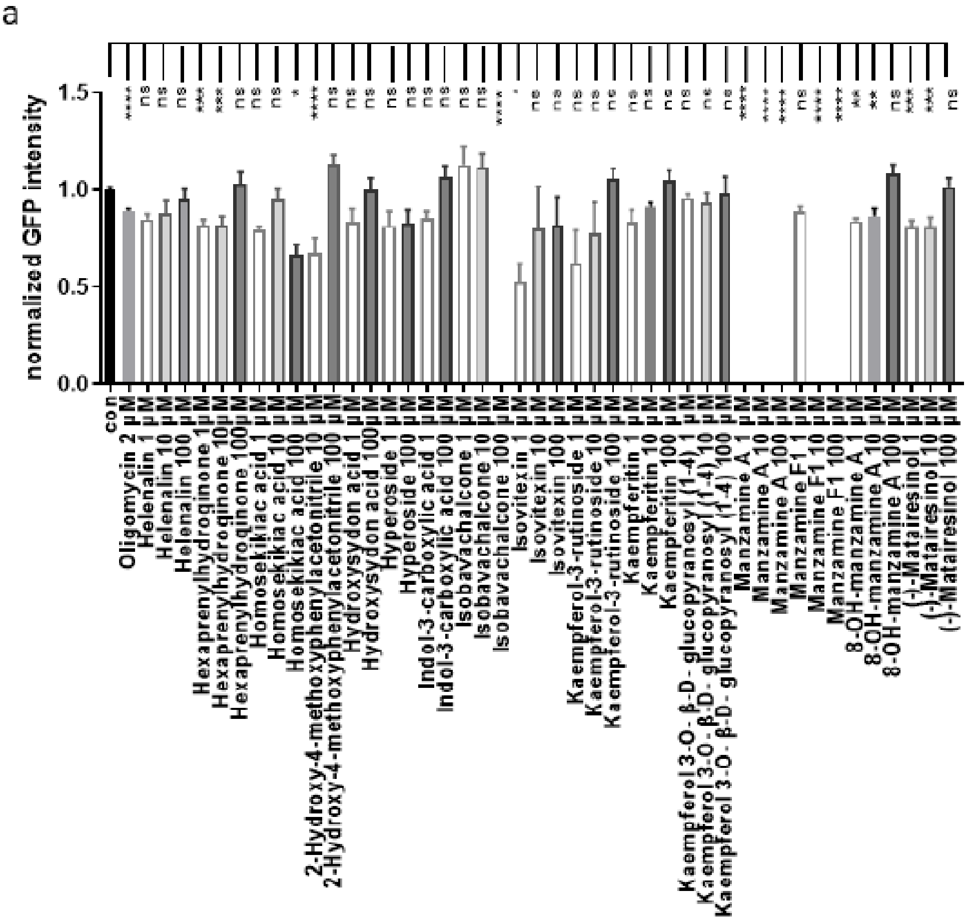

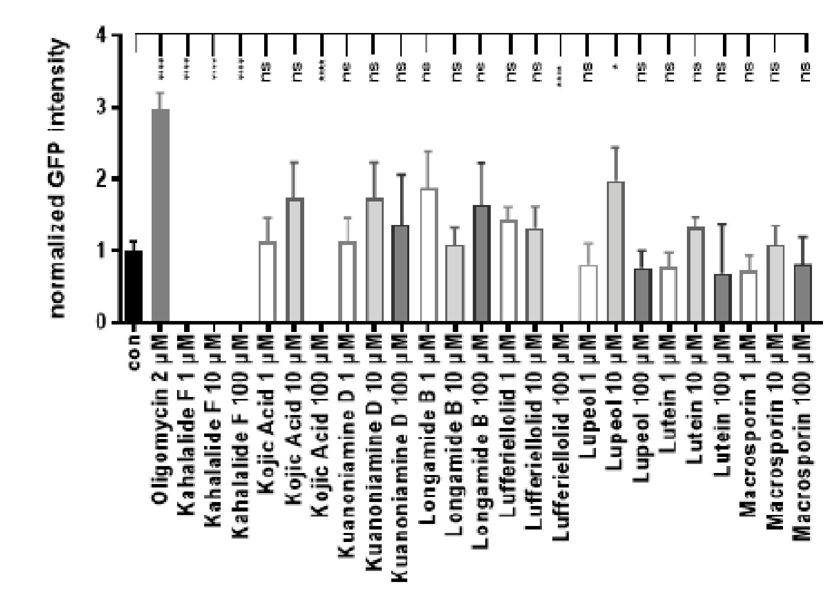

Figure S2. Fluorescence intensity quantified with the Cellomic ArrayScan reader in liquid culture. In a) the *C. elegans* strain used for the screening was a transcriptional reporter for gst-4 and in b) a transcriptional reporter for hsp-6. The results highlight few compounds with the ability to significantly alter the GFP expression. The GFP intensity was normalized to 1 for control worms, treated with 1% DMSO. Bar graphs represent means ± SEM. Asterisks (*) denote significant differences vs control, unpaired t-test. *p-value <0.05 ** p value < 0.01, ***p-value < 0.001, ****p-value < 0.0001; ns stands for not significant.

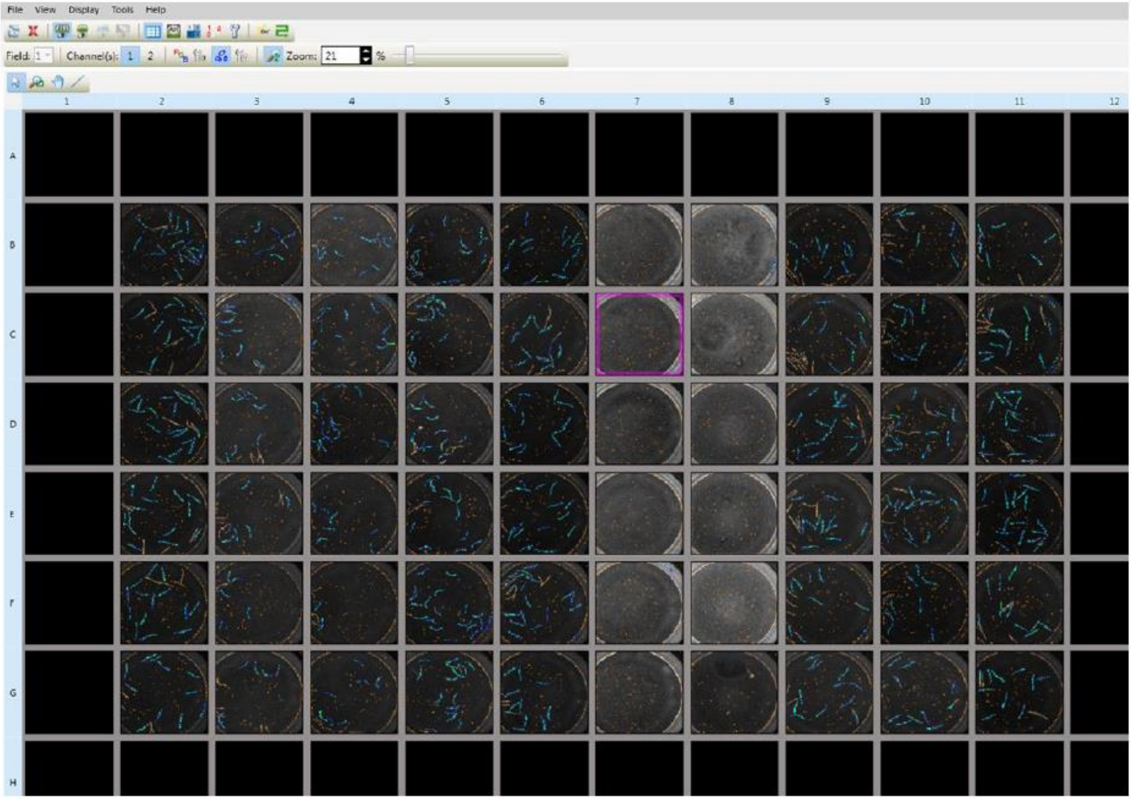

Figure S3. Representative picture of a 96-well plates scanned with the ArrayScan in liquid culture. Blue tagged worms are identified as objects, and GFP is visible in green. Excluded objects are tagged in orange. The wells of each column contain worms treated with one of the compounds under study at one specific concentration. The pink square highlights which well is selected by the user at the moment in which the picture was taken, right clicking allows to visualize details for the selected well.

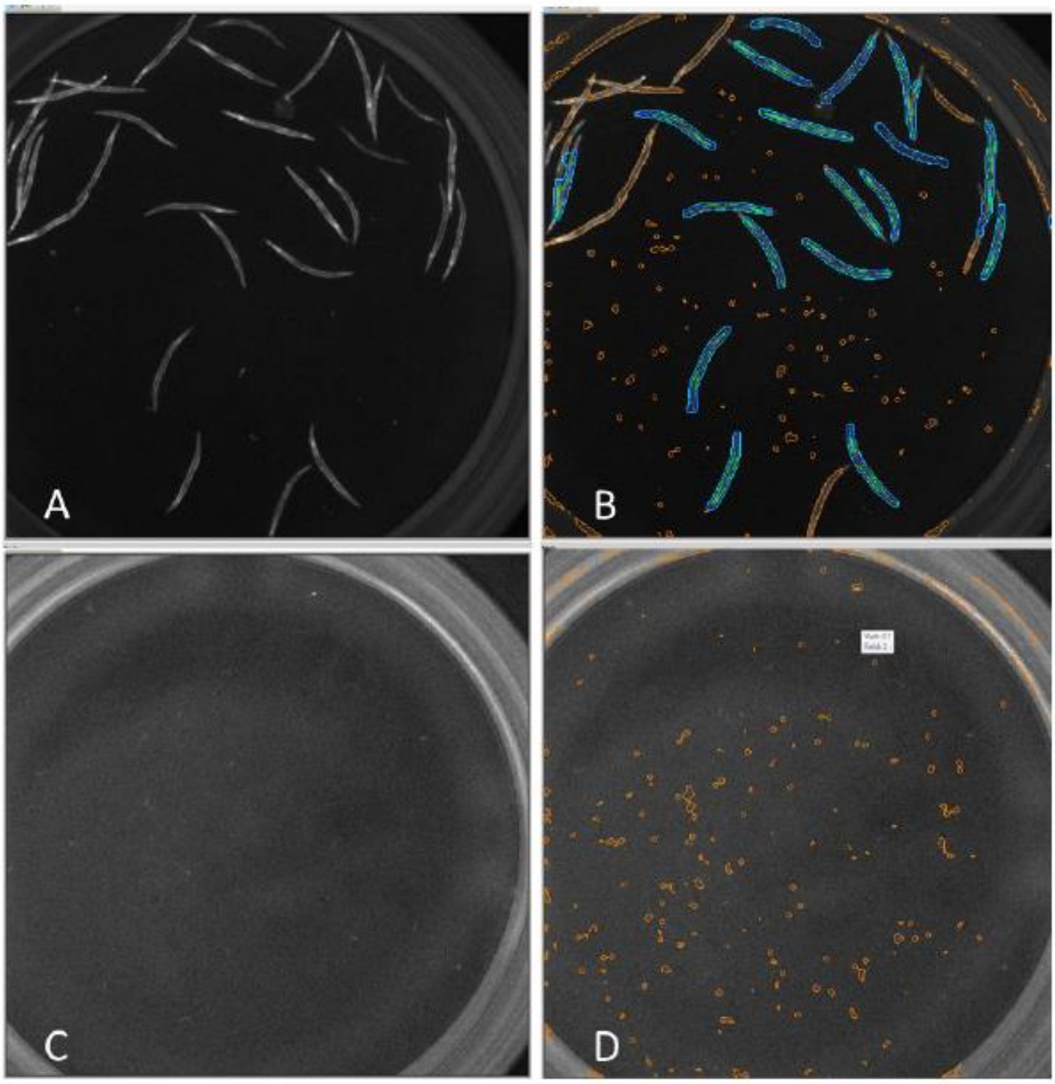

Figure S4. Representative wells from a scanned plate in liquid culture. A-B) Particular of 2 wells in which the animals are identified as valid objects are marked with a blue mask and the one excluded are marked orange. In panel C the worms are too small and no object is identified, in D all objects are excluded.

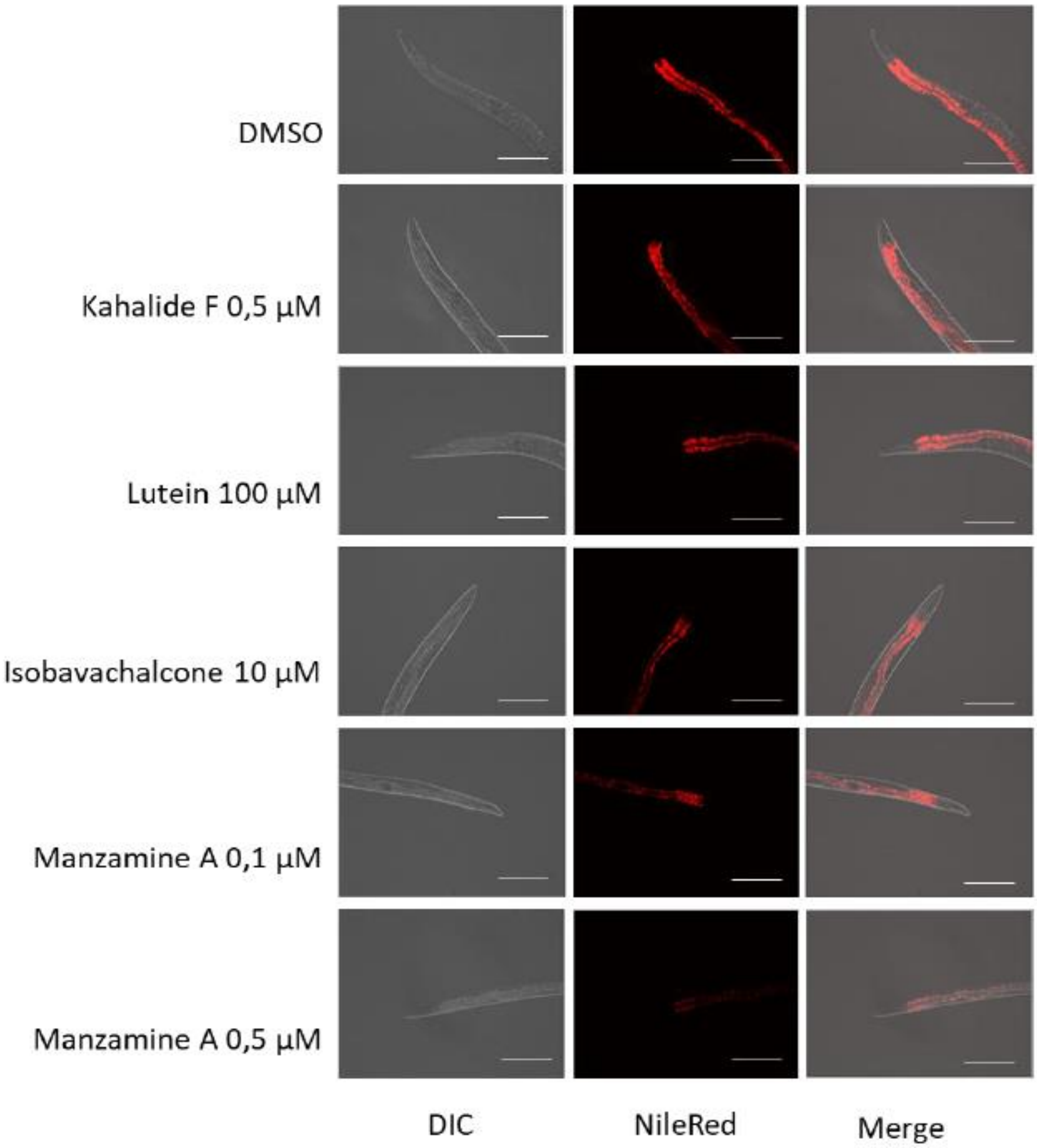

Figure S5. Representative images of Nile Red fluorescence of nematodes. Animals were treated with the indicated compounds, on solid media, from eggs. Pictures were acquired on the first day of adulthood. Nematodes were grown in presence of Nile Red (in the dark) from eggs to gravid adult stage. Scale bar 200 µm.

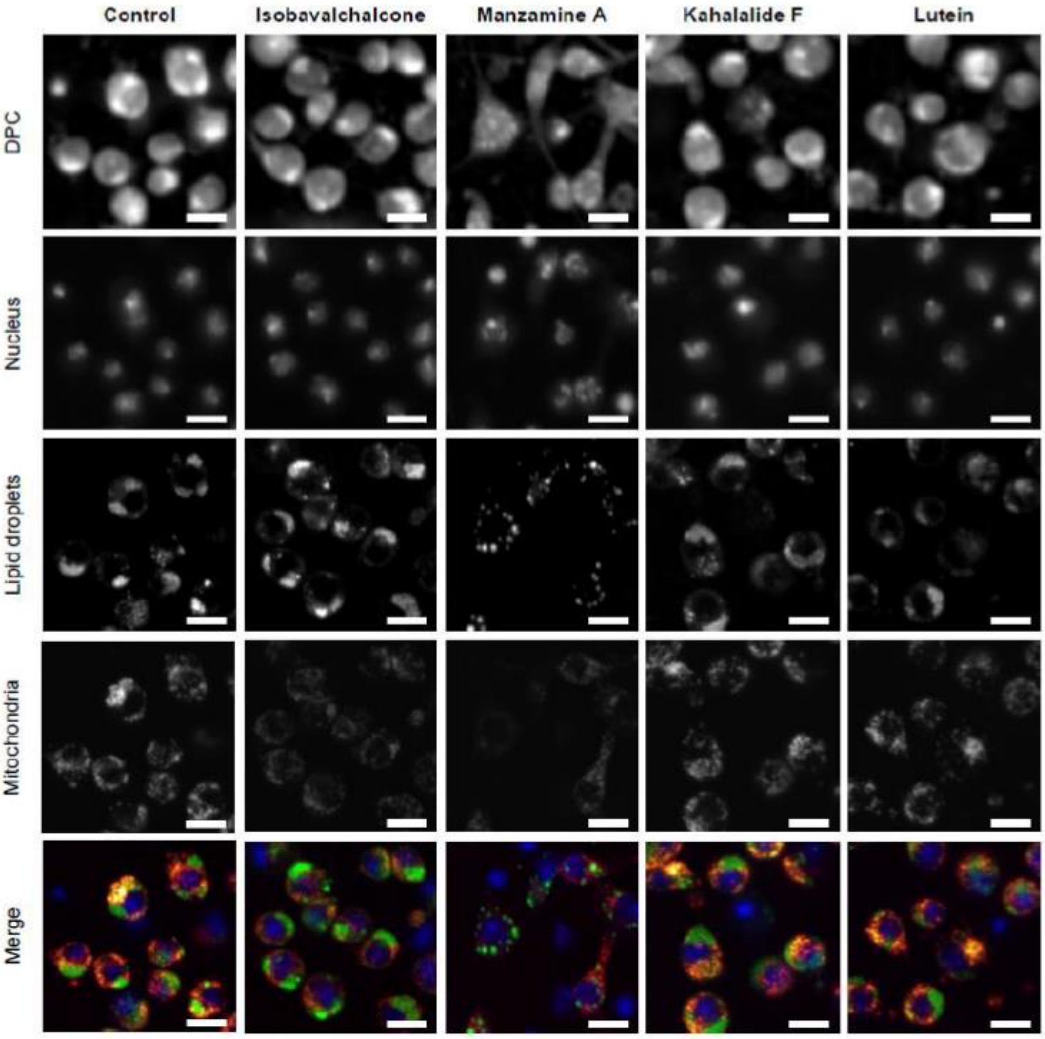

Figure S6. High content microscopy assay with *Drosophila melanogaster* S2R+ cells. Representative picture of the high content staining in control and isobavachalcone, manzamine A, kahalalide F and lutein (all four at 20 μM) treated cells. S2R+ cells were incubated for 18 hours with the different concentrations of the four hit compounds. The control group was treated with DMSO (0.2 %). Merge is from nucleus (blue, Hoechst 33342), lipid droplets (green, BODIPY FL C12) and mitochondria (red, TMRE). Digital phase contrast is calculated from transmitted light images and shows the unstained cell morphology. Scale bar = 10 μm.

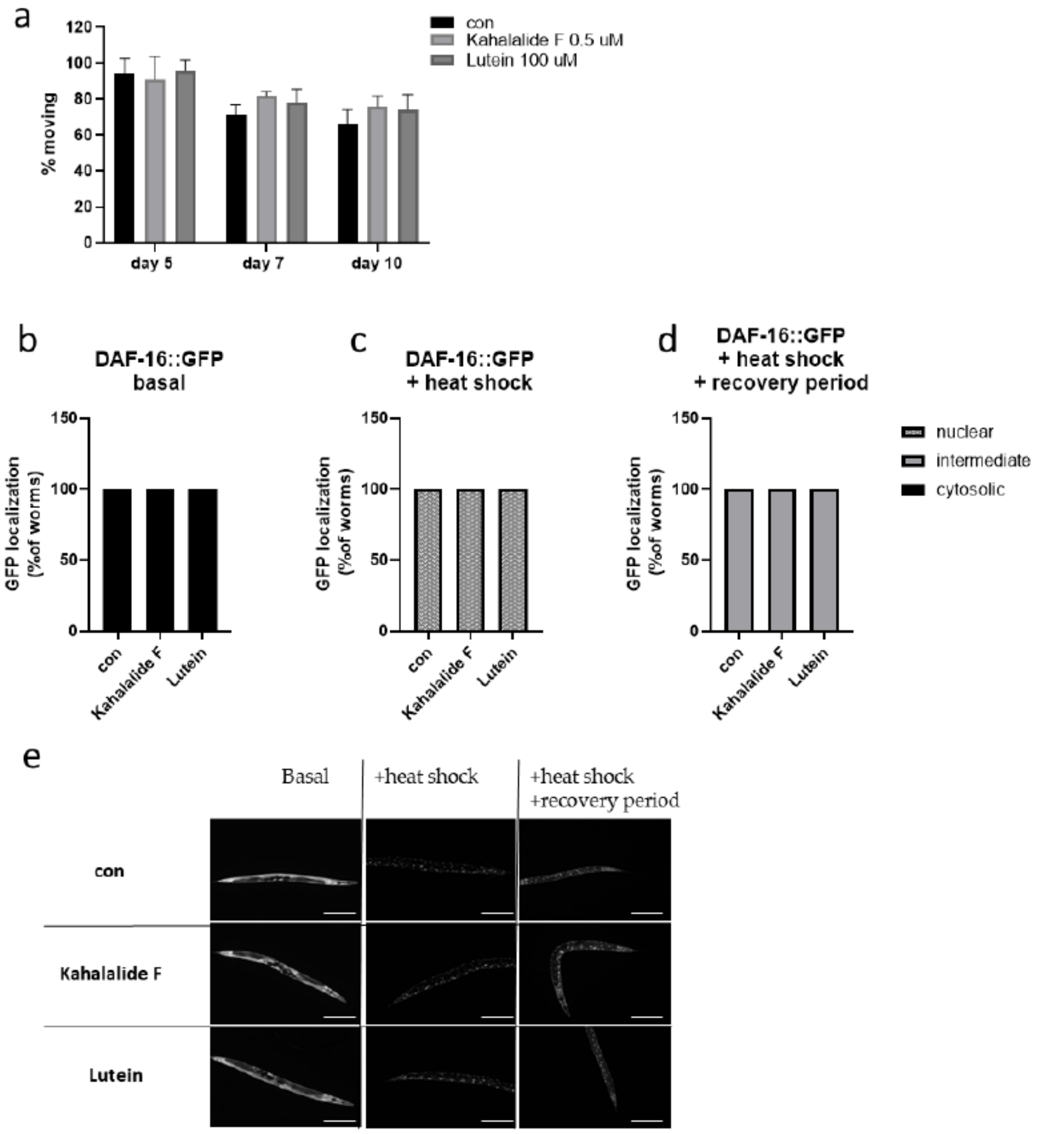

Figure S7. Modulation of movement and heath shock-induced DAF-16 upon lutein and kahalalide F treatments. a) Health-span of nematodes treated with vehicle, kahalalide F or lutein, calculated as % of moving animals (among surviving worms) over time. Three time points during animals' lifespan were chosen. b-d) DAF-16::GFP nuclear translocation in basal conditions (b), upon one hour at 35°C, heat-shock (c) and after 2 hours of recovery period (d). Lutein and kahalalide F did not affect DAF-16 translocation. e) representative pictures of DAF-16 reporter strain showing the nuclear translocation in basal condition, under heat-shock and after 2 hours of recovery period after stress. Scale bar 200 µm.

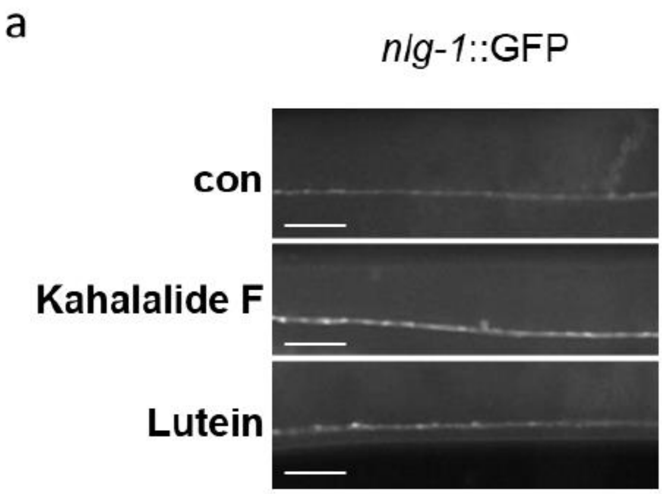

Figure S8. ***nlg-1*** **induction upon Lutein and kahalalide F treatments.** a) Representative pictures of P*nlg-1*::GFP expression in ventral nerve cord. Scale bar 20 μm. Quantification of GFP signal is shown in Fig 6d.
